# Ancient but Not Forgotten: New Insights Into MPEG1, a Macrophage Perforin-Like Immune Effector

**DOI:** 10.3389/fimmu.2020.581906

**Published:** 2020-10-15

**Authors:** Charles Bayly-Jones, Siew Siew Pang, Bradley A. Spicer, James C. Whisstock, Michelle A. Dunstone

**Affiliations:** ^1^ARC Centre of Excellence in Advanced Molecular Imaging, Monash University, Melbourne, VIC, Australia; ^2^Department of Biochemistry and Molecular Biology, Biomedicine Discovery Institute, Monash University, Melbourne, VIC, Australia; ^3^John Curtin School of Medical Research, Australian National University, Canberra, ACT, Australia

**Keywords:** MACPF/CDC, MACPF domain, pore-forming protein, immune effector, immunology, PRF2, MPEG1

## Abstract

Macrophage-expressed gene 1 [MPEG1/Perforin-2 (PRF2)] is an ancient metazoan protein belonging to the Membrane Attack Complex/Perforin (MACPF) branch of the MACPF/Cholesterol Dependent Cytolysin (CDC) superfamily of pore-forming proteins (PFPs). MACPF/CDC proteins are a large and extremely diverse superfamily that forms large transmembrane aqueous channels in target membranes. In humans, MACPFs have known roles in immunity and development. Like perforin (PRF) and the membrane attack complex (MAC), MPEG1 is also postulated to perform a role in immunity. Indeed, bioinformatic studies suggest that gene duplications of MPEG1 likely gave rise to PRF and MAC components. Studies reveal partial or complete loss of MPEG1 causes an increased susceptibility to microbial infection in both cells and animals. To this end, MPEG1 expression is upregulated in response to proinflammatory signals such as tumor necrosis factor α (TNFα) and lipopolysaccharides (LPS). Furthermore, germline mutations in MPEG1 have been identified in connection with recurrent pulmonary mycobacterial infections in humans. Structural studies on MPEG1 revealed that it can form oligomeric pre-pores and pores. Strikingly, the unusual domain arrangement within the MPEG1 architecture suggests a novel mechanism of pore formation that may have evolved to guard against unwanted lysis of the host cell. Collectively, the available data suggest that MPEG1 likely functions as an intracellular pore-forming immune effector. Herein, we review the current understanding of MPEG1 evolution, regulation, and function. Furthermore, recent structural studies of MPEG1 are discussed, including the proposed mechanisms of action for MPEG1 bactericidal activity. Lastly limitations, outstanding questions, and implications of MPEG1 models are explored in the context of the broader literature and in light of newly available structural data.

## Introduction

The superfamily of MACPF/CDCs consists of proteins ubiquitously found in many kingdoms of life, including bacteria, plants, fungi and animals. Collectively, these molecules perform diverse functions, including roles in plant defence (*e.g. Arabidopsis thaliana*; CAD1, NSL1) (1–3), microbial virulence (Gram-positive bacteria; CDCs) (4–6), ingress and egress (*e.g. Plasmodium* spp.; SPECT, MAOP) (7, 8), as venom components (*e.g.* sea anemone toxin PsTX-60B, stonustoxin, perivitellin-2) (9–12), and nutrition (*e.g.* fungi; pleurotolysin) (13, 14). In humans MACPF proteins play key roles in immunity and development [*e.g.* mammalian perforin (PRF) and complement component 9 (C9)] (15–18).

The first indirect observations of a MACPF pore-forming complex were made by Jules Bordet in 1898 (19). Here, it was observed that certain blood factors complemented antibodies in their function and could lyse red blood cells. These early studies led to the identification of the Membrane Attack Complex (MAC), which is the terminal effector of the complement pathway (16). The MAC is formed *via* the recruitment of C5, C6, C7, C8, and, finally, multiple copies of C9 (20–23). The latter protein forms a transmembrane pore that can function in immunity to clear a wide variety of invading microorganisms including gram-negative bacteria (24–27), protozoa, enveloped viruses, and helminths (28, 29). These microbes can be eliminated by either direct lysis of cells or *via* facilitating the entry of other immune effectors (30, 31). Formation of sub-lytic levels of the MAC on local host cells also triggers cellular signal transduction pathways to control local inflammatory and immune responses (32–34). Loss of MAC activity can lead to recurrent infections (35, 36), while aberrant and excessive MAC activity can lead to life-threatening disease, such as paroxysmal nocturnal hemoglobinuria, where red blood cells become lysed due to unregulated MAC activity (37, 38).

A second immune pore-forming MACPF protein, perforin (PRF), was identified in the mid 1980s through the work of Podack, Tschopp and colleagues (39, 40). PRF is held inside of granules within cytotoxic T lymphocytes and natural killer cells (40). Upon formation of the immune synapse with a virally infected or transformed cell, PRF is secreted into the synapse, whereupon it oligomerizes to form pores in the target cell membrane (41–43). These pores permit the translocation of granzymes (which are secreted with PRF) into the target cell, an event that results in apoptosis (44, 45). Complete loss of PRF results in familial hemophagocytic lymphohistiocytosis type II, where expansion of antigen presenting cells becomes uncontrolled as normal apoptotic clearance is disrupted (46). This disease manifests itself in individuals as high levels of circulating active leukocytes, which secrete proinflammatory cytokines resulting in fever and a cytokine storm which, if untreated, ultimately leads to patient death (46, 47).

Outside the realm of immunology, several MACPFs appear to govern developmental processes within humans, namely the astrotactins (ASTN1 and ASTN2) and the bone morphogenetic protein/retinoic acid inducible neural-specific proteins (BRINP1, BRINP2 and BRINP3) (48). While the biological functions of the ASTNs and BRINPs are unknown, they have been implicated in brain development. Specifically, ASTNs are involved in neuronal migration and glial cell adhesion (49–51). Conversely the roles of BRINPs are poorly understood, with some suggestions that they function in neuroplasticity or regulating cell cycles (52, 53). Mutations in both ASTNs and BRINPs have been associated with intellectual disabilities, ADHD, and other neuropathies (54–56). It is currently unclear whether ASTNs or BRINPs form pores as part of their function.

An intriguing family of pore-forming proteins (PFPs), the gasdermins (GSDMs), represents a class of intracellular immune effectors which like MACPFs form giant *β*-barrel pores (57, 58). Specifically cleaved and activated by caspases during microbial infection or after detection of danger signals, GSDMs are converted from an autoinhibited state into two fragments (59). Cleaved-GSDMs rapidly form pores in the cell membrane (from the cytosolic side) and are considered effectors of cellular pyroptosis, a form of programmed cell death (60, 61). The GSDM fold is suggested to define a unique family, although striking similarities with MACPF proteins have been noted (62, 63).

The primary topic of this review, MPEG1, was identified through the *in situ* analysis of differential gene expression comparing mature with immature cell lines of mouse macrophages (64). Numerous names for this pore-forming protein have since arisen, including macrophage-expressed gene 1 (MPEG1 or sometimes MPG1), perforin-2 (PRF2 or P2) and macrophage specific gene 1 (MSP1). We elect to use the original name, MPEG1, owing to its widespread use and to distinguish MPEG1 from PRF. MPEG1 is postulated to represent the closest paralog of the common MACPF ancestor gene in metazoa (65, 66). Bioinformatic analyses reveal that complete MPEG1 homologs can be found throughout the kingdom *Animalia* with exemplars identifiable in phyla including sea sponges (Porifera), sea anemones (Cnidaria) (67), comb jellies (Ctenophora) (68), flatworms (Platyhelminthes) (69), molluscs (Mollusca) (70–76) and all chordate organisms (66, 77). These studies also suggest that gene duplications of MPEG1 likely gave rise to the mammalian immune effectors PRF and the terminal components of the complement system (MAC), which are observed after notochord evolution. Resultantly, MPEG1 represents the earliest known and most ancient MACPF protein identified to date in metazoan immune and defense systems (66, 78).

From the structural perspective, MPEG1 is an intracellular type-1 transmembrane MACPF protein with both a vacuole-based ectodomain and cytosolic region (endodomain). MPEG1 traffics *via* the ER, Golgi, and secretory vesicles to localize to early endosomes and phagosomes/lysosomes (79–81). It is thought that MPEG1 functions in the phagosome in an anti-microbial capacity. Furthermore, recent structural studies strongly support the idea that MPEG1 is a *bona fide* pore-forming immune effector. In this regard, early electron-microscopy (EM) studies revealed that MPEG1 is able to form oligomers of a size and shape typical for members of the MACPF/CDC superfamily (79). The first three-dimensional structure of human MPEG1 revealed the molecule was able to assemble into an oligomeric prepore intermediate, and that this material was able to form pores upon acidification (82). This is consistent with MPEG1 performing a role in the phagosomal system. Indeed, analysis of the primary structure of MPEG1, together with imaging data, suggests that the pore-forming machinery of the MPEG1 MACPF domain is positioned within the luminal environment of the membrane vesicles (78–81). Consistent with these data, a low-resolution detergent solubilized structure of murine MPEG1 suggests the molecule can form membrane spanning pores (83). Finally, germline mutations in MPEG1 have been found in patients suffering from recurrent pulmonary non-tuberculous mycobacterial infections (84). Thus, the available data to date suggests that MPEG1 is an immune effector involved in defense against invading intracellular pathogens and processing of engulfed pathogens. Overall, several mechanisms are proposed to explain MPEG1 mode of action *in vivo*.

In this review, we discuss the current understanding of MPEG1 evolution, regulation, and structure–function. We furthermore evaluate the contrasting proposed mechanisms of bactericidal activity. Taken together, we explore the implications of cellular and whole-organism research in the context of new MPEG1 structure–function studies and discuss the outstanding questions surrounding this ancient immune effector.

## Overall Domain Architecture of MPEG1

**Figure 1:**
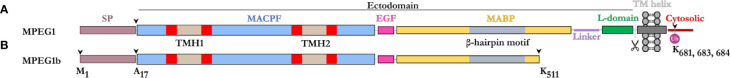
Domain schematic of MPEG1 in two isoforms. **(A)** The majority of MPEG1 consists of a MACPF (blue) and MABP (yellow) domain. The MACPF domain contains functional motifs, namely the TMH regions (tan/red) that unfurl to form a *β*-barrel upon pore formation. The MABP domain contains a *β*-hairpin motif (gray) that recognizes and binds to negatively charged phospholipids. A small EGF-like motif (pink) is located between the MACPF and MABP domains. The MABP domain is followed by a small linker region (purple) and conformationally labile motif, denoted the L-domain (green). These directly precede the transmembrane helix (dark gray) and cytosolic (red) regions. The cytosolic region contains a lysine rich motif that is monoubiquitinated during immune response. Scissors depict the putative cleavage site of MPEG1. The majority of MPEG1 constitutes the ectodomain and is postulated to be proteolytically shed from the bilayer. **(B)** MPEG1b is a shorter secreted isoform that is truncated in the MABP domain at K511 (arrow). Both MPEG1 and MPEG1b are directed to the ER by a signal peptide (SP; copper rose) that is cleaved (arrow) shortly after translation.

The majority of the MPEG1 sequence forms an ectodomain component, which comprises an N-terminal MACPF domain, followed by a multi-vesicular body-12 (**M**VB12)-**a**ssociated *β*-prism (MABP) domain (**Figure 1**). A signal peptide (SP) precedes the ectodomain and directs MPEG1 to the endoplasmic reticulum (ER) (**Figure 1**). While the SP is proteolytically removed shortly after translation, it positions the MPEG1 ectodomain within the lumen of the ER. Between the MACPF and MABP domains, MPEG1 also possesses a small folded linker region which is postulated to be an EGF-like domain (**Figure 1**). Furthermore, a second small folded region exists between the MABP domain and the Type I transmembrane helix of unknown fold named either the L-domain (82) or CTT (83) (**Figure 1**). Directly after the L-domain there follows a single pass type I transmembrane helix and a cytosolic region (**Figure 1A**).

A shorter isoform of MPEG1 has also been described (81), whereby alternative splicing gives rise to a product that is truncated at residue K511 within the MABP domain (**Figure 1B**). This shorter isoform, named MPEG1b (or PRF2b), therefore lacks a part of the MABP domain and the entire transmembrane and cytosolic regions. For the purposes of this review, when referring to the shorter isoform we will use MPEG1b, otherwise MPEG1 is used to denote the full-length isoform.

Prior to its structural characterization (82), the MPEG1 MABP domain was referred to as the P2 domain (for PRF2 domain), since the homology with the MABP fold could not be identified through sequence analysis alone (79). The MPEG1 MABP domain appears to be somewhat divergent, possessing an inserted hyper-extended *β*-hairpin motif, which likely explains why sequence analysis failed to identify the domain. Previously, MABP domains have been implicated in lipid binding (85, 86).

## MPEG1 Expression and Regulation in the Cellular Context

**Figure 2:**
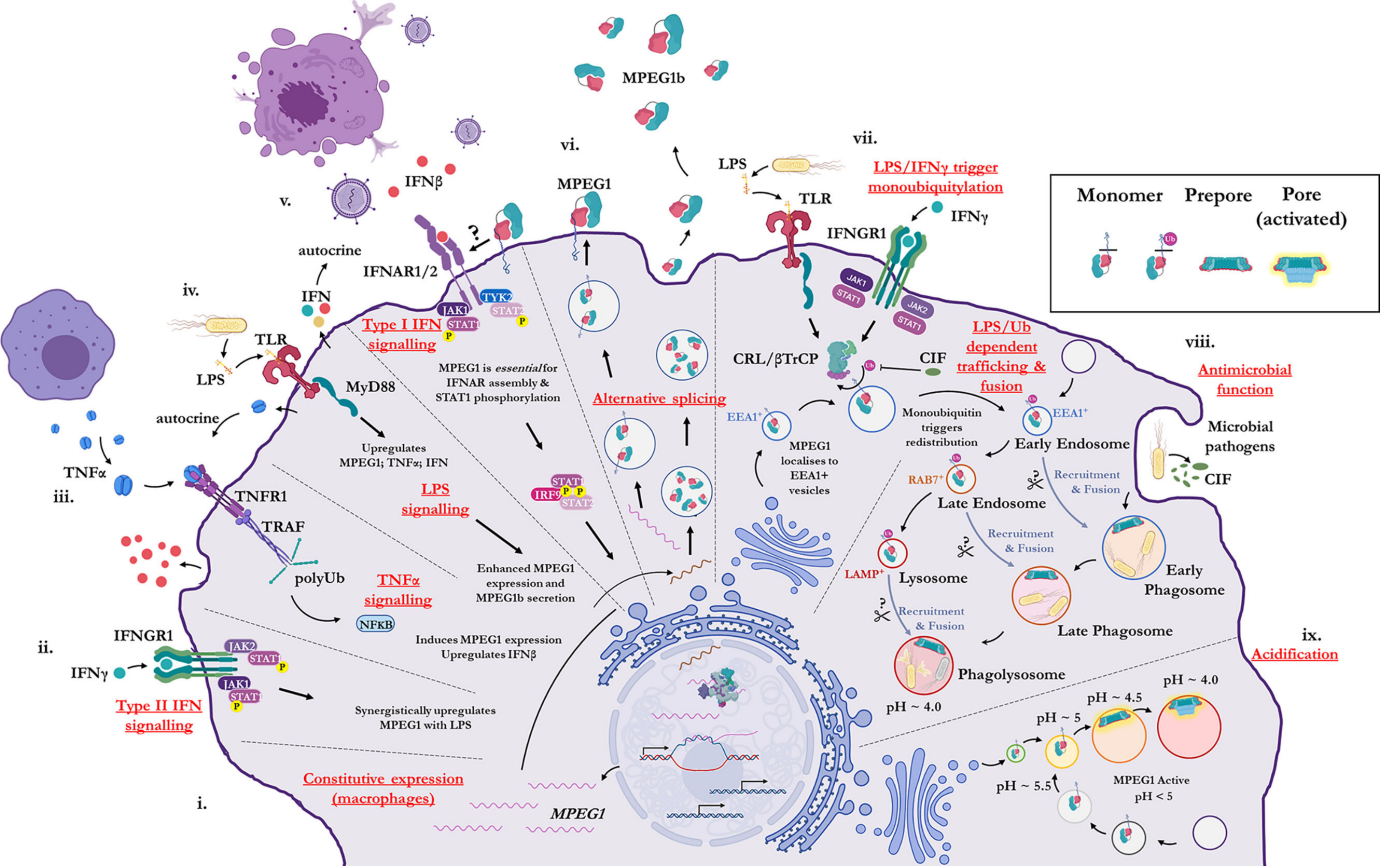
Illustration of the cellular context and key processes in which MPEG1 is implicated. **(i)** MPEG1 is constitutively expressed in macrophages and phagocytes. **(ii, iii, iv)** Type II IFN, TNFα and LPS signaling are involved in the regulation of MPEG1 expression. **(v)** Surface bound MPEG1 is essential for the correct assembly and signaling of the type I IFN pathway. **(vi)** MPEG1 is expressed either as a membrane tethered isoform (MPEG1) or as a secreted, truncated form (MPEG1b). **(vii)** LPS and IFN*γ* signaling are important for triggering monoubiquitination of MPEG1 in EEA1^+^ vesicles. Some pathogens produce CIF which inhibits the monoubiquitination of MPEG1 and confers resistance. **(viii)** Once monoubiquitinated, EEA1^+^/MPEG1^+^ vesicles traffic to and fuse with the early phagosome. The phagocytic and secretory pathways cooperate to enrich intracellular compartments with MPEG1 to aid in the disruption and killing of engulfed microbes. Proteases (scissors) may be important for MPEG1 function. **(ix)** As phagosomal and endosomal vesicles become acidified, MPEG1 is activated to form lytic pores. Figure produced with BioRender.

Historically, MPEG1 was discovered in macrophages; however, it is now clear that many cell types can express MPEG1 (79). Constitutive MPEG1 expression is common in macrophages and leukocytes (**Figure 2i**); however, others have observed that expression can be induced in a wide variety of other cell types such as epithelial and fibroblast lines (79, 87) (**Figures 2ii–iv**). Furthermore, proinflammatory signals were observed to trigger the upregulation of MPEG1 expression (**Figures 2ii–v**). For example, parenchymal, epithelial, and fibroblast cells, which usually do not express MPEG1, can be induced to express MPEG1 upon stimulus during an inflammatory response (**Figures 2ii–v**) (79). In this regard, tumor necrosis factor α (TNFα) and lipopolysaccharide (LPS) signaling have been shown to drive the expression of MPEG1 independently. Hence, MyD88 and NF*κ*B pathways have been implicated for MPEG1 expression (65, 88). When used in combination, TNFα and LPS were observed to have an additive effect upon MPEG1 gene expression. Similarly, interferon (IFN)-*γ* upregulates MPEG1 expression synergistically with LPS, while IFN*γ* alone has no effect (81) (**Figures 2ii–iv**). In addition to these effects, in the context of MPEG1b, stimulation of cells by LPS alone also triggered enhanced secretion of MPEG1b (81) (**Figure 2iv**).

MPEG1 is expressed as a type-I transmembrane protein where, upon translation, the C-terminal transmembrane helix anchors MPEG1 into the bilayer (**Figure 1A**; **Figure 2vi**). As such, the folded ectodomain of MPEG1 is located on the luminal side of the ER with the cytoplasmic tail protruding into the cytoplasm. The cytoplasmic tail and transmembrane helix act to facilitate correct trafficking *via* the secretory pathway whereby MPEG1 migrates through the ER, Golgi, *via* secretory vesicles to fuse and accumulate within early endosome antigen 1 (EEA1+) vesicles (79) (**Figure 2vii**). MPEG1 is also found associated with the plasma membrane, with the ectodomain oriented into the extracellular space (81, 89) (**Figure 2vi**), where surface bound MPEG1 is implicated in type I IFN signaling (89) (**Figure 2v**). Conversely, since MPEG1b lacks the transmembrane domain, this short isoform is not membrane tethered and is therefore secreted into the extracellular space (**Figure 2vi**). While MPEG1 likely functions in a bactericidal capacity, the function of extracellular MPEG1b remains poorly characterized.

## LPS/IFNγ Dependent Monoubiquitination and Translocation

In addition to stimulating expression, proinflammatory stimuli prompt the redistribution and translocation of MPEG1 (**Figure 2vii**) (80). Specifically, LPS and type II interferon signaling lead to post translational modification of the MPEG1 cytosolic region (**Figure 2vii**). Upon engulfing microbes, cells are stimulated by LPS and IFN*γ* which initiates monoubiquitination of MPEG1 by a Cullin-RING ligase (CRL) and *β*-Transducin Repeat Containing Protein (*β*TrCP) complex (**Figure 2vii**). A conserved, lysine rich region (K681, K684, K685) located in the cytosolic tail of MPEG1 (**Figure 1A**), is resultantly modified by a monoubiquitin. When monoubiquitinated, EEA1^+^/MPEG1^+^ vesicles traffic and fuse with LAMP^+^ vesicles (late phagosomes, phagolysosomes and/or lysosomes) (80, 81) (**Figure 2viii**). These cellular re-distribution events ultimately result in MPEG1 co-localization with phagosomal vesicles containing engulfed microbes (79–81) (**Figures 2vii–ix**).

This re-organization is paramount for the function of MPEG1. Mutation of the lysine rich region produces a form of MPEG1 that prevents trafficking and accordingly a null phenotype (80). Indeed, certain intracellular pathogens produce molecules (CIF; cycle inhibiting factor) that inhibit the ubiquitination machinery of mammalian cells, thus disrupting the post translational modification of MPEG1 and therefore preventing further maturation and trafficking (80) (**Figure 2viii**). It is, therefore, unsurprising that inhibition of MPEG1 trafficking confers resistance to microbes, such as enteropathogenic *Escherichia coli* and *Yersinia pseudotuberculosis*, from the host cell (**Figures 2vii, viii**). Hence, the cytosolic tail functions as a signal-dependent trafficking motif. This implies monoubiquitination-dependent trafficking of MPEG1b cannot occur, since it lacks the signaling motif present in the cytosolic tail. Thus, external stimulation from microbes by proinflammatory molecules coordinates the re-organization of MPEG1 in preparation for phagocytosis.

## MPEG1 Function in Immunity

The available evidence to date suggests that MPEG1 functions as an immune effector. Despite the significance of MPEG1 as a likely ancestor to the better-known immune effectors C9 and PRF, its precise function has remained poorly understood. However, given the role of the MAC and PRF, and the localization of MPEG1 to macrophages, it was suggested that MPEG1 may perform an anti-microbial role. Indeed, recombinantly produced sponge MPEG1 and the MACPF domain of oyster MPEG1, have anti-microbial activity *in vitro* (65, 72). MPEG1 expression is induced by proinflammatory signals in several dozen cell lines and, furthermore, multiple cell lines succumb to bacterial infection when MPEG1 is knocked-down or knocked-out (79–81, 90, 91). In vertebrates, MPEG1-deficient mice and zebrafish are more susceptible to infections (by Methicillin-resistant *Staphylococcus aureus*, *Salmonella typhimurium* and *Mycobacterium marinum*) compared to wild-type counterparts (88, 90). Likewise, several studies have illustrated MPEG1 antibacterial activity in invertebrates (67, 68, 70–74).

Recombinant forms of MPEG1 from sponge (65), oyster (MACPF domain only) (72) and fish (92) were all observed to be bactericidal *in vitro*. When challenged by *M. marinum*, one of the three MPEG1 paralogs in zebrafish (*mpeg1.2*) becomes upregulated. Of the remaining two, one is thought to be a pseudogene (*mpeg1.3)*, while the other (*mpeg1)* is surprisingly suppressed. Knock-down experiments of *mpeg1.2* resulted in an increased bacterial burden on zebrafish challenged with *M. marinum*, while knock-down of *mpeg1* gave a survival advantage compared to wild type fish, suggesting an altered immune response (88). Several studies of MPEG1 in vertebrates and invertebrates now support the model that suppression or complete loss of MPEG1 results in a loss of bactericidal activity. Collectively, these studies demonstrate the essential role MPEG1 plays as an immune effector against microbes and bacteria.

In one experiment McCormack and colleagues observed *Mycobacterium smegmatis* swelling (albeit not killing) after treatment with MPEG1. The addition of minute quantities of lysozyme, however, were sufficient to kill pre-treated *M. smegmatis*, putatively indicating MPEG1 perforates the outer membrane but does not affect the integrity of the peptidoglycan layer (90). Indeed, more recent studies have found MPEG1 facilitates the entry of myriad anti-microbial effectors into cells including; proteases, reactive oxygen and nitrogen species, bactericidal peptides and the harsh acidic environment of the phagosome (93). Notably, recombinant MPEG1 possesses lytic activity which is strictly dependent on low pH (82, 83). Lastly, MPEG1 has been observed to form membrane spanning pores similar to the lytic MAC and PRF (13, 94).

To date, no severe disease state has been described in humans with complete loss of MPEG1 function. Like many aspects of biology, there may be compensating redundancies in the immune system that maintain a sufficient immune response in the absence of MPEG1. One retrospective case study, however, has described recurrent pulmonary non-tuberculous mycobacterial infections in individuals with germline mutations in MPEG1 (84). The association of these mutations with recurrent infections suggests there may be subtle clinical outcomes in individuals with a defective form of MPEG1; however, further clinical data is required to definitively implicate MPEG1 in these pathologies. Furthermore, there are presently no examples of excessive MPEG1 function, which contrasts to MAC and PRF where hyperactivity can lead to severe disease states in humans (47, 95, 96).

## Interferon Signaling/LPS Induced Shock

Apart from anti-microbial function by pore formation, MPEG1 has been implicated in regulating type I IFN signaling and is critical for the correct assembly and signaling of the Interferon-*α*/*β* receptor (IFNAR) proximal complexes (89) (**Figure 2v**). Transfection studies, using several MPEG1 truncation variants, illustrate the assembly of proximal complexes and phosphorylation of downstream signaling effectors are dependent on MPEG1. Type I IFN signaling was found to be defective in MPEG1-deficient cell lines (BMDM, MEFs) due to loss of IFNAR mediated phosphorylation of STAT1, STAT2, JAK1, and TYK2. It was observed that both the MACPF and MABP domains appear to mediate interactions with IFNAR1 and IFNAR2, respectively, while the intracellular cytosolic region was required for phosphorylation of STAT2. Currently, however, the nature of MPEG1–IFNAR interactions is poorly understood.

Animal models have shed light on the MPEG1–IFNAR association and the significance of this poorly understood signaling pathway. Under normal circumstances excessive LPS can overwhelm the host immune response and lead to septic shock. Mice lacking MPEG1 were found to be resistant to LPS induced septic shock (89). Consistently, the suppression of an MPEG1 paralog in zebrafish was observed to reduce the likelihood that fish would succumb to infection (88). Taken together, these studies suggest MPEG1 plays a role in the excessive IFN signaling during an overwhelming immune response.

Overall, these findings suggest MPEG1 is not only important for cellular immunity, but also the regulation of immune response *via* IFN*α*/*β* signaling. This has implications for both autoimmune disorders and cancer. In a recent example studying MPEG1-deficient, aged mice, there was an increased proportion of microbial migration into serum from the gastrointestinal tracts, which resulted in a state of chronic inflammation (97). A significantly reduced antibody response was also observed in these MPEG1-deficient mice. Furthermore, an increased level of inflammation was observed in splenic B cells, as well as an increased frequency of pro-inflammatory B cells. These data suggest that an increased bacterial burden in those who lack MPEG1 may be responsible for chronic inflammation, which is proposed to affect the normal immune responses of B cells. Chronic inflammation is a known determinant of many autoimmune diseases, and hence, reduced levels of MPEG1 may also contribute to a number of chronic, progressive diseases in humans.

## MPEG1 Proteolysis

Proteolytic shedding of MPEG1 has been proposed for proper MPEG1 regulation and function. It is hypothesized that proteolysis may be required for the release of the ectodomain from the host membrane to allow for pathogen targeting and pore-forming function. Proteolytic processing may function two-fold to regulate MPEG1 function. Firstly, proteolysis may be necessary for oligomerization to occur (79). Secondly, anchoring MPEG1 to the host bilayers (endosome, secretory vesicles, *etc*.) may prevent unintended lytic function. Lastly, sequestration of MPEG1 by the transmembrane region is important for trafficking (81, 89). Therefore, untimely proteolytic processing may result in MPEG1 mis-trafficking (**Figure 2viii**).

In the context of invading microbes, fragments of MPEG1 corresponding to the ectodomain (among others) have been identified in bacterial membranes treated with MPEG1 (79). Notably, the C-terminal cytosolic region was not detectable, consistent with suggestions that the transmembrane and cytosolic tail remains in the host membrane. Furthermore, limited proteolysis experiments of MPEG1-enriched bilayers derived from transfected cells resulted in membrane-bound ring-like oligomers (79), whereas, these oligomers were not observed in the absence of proteolytic treatment. This supports the current model whereby the ectodomain is shed during MPEG1 anti-microbial function.

These findings suggest that spatio-temporal regulation of MPEG1 proteolysis is associated with normal function. The mechanism of this proteolysis is still being determined and the endogenous protease responsible for MPEG1 cleavage has not been identified. Further studies will be required to confirm the sites of MPEG1 cleavage and their functional role within the cellular context. While the relevance and functional importance of MPEG1 proteolysis is unclear, it is notable that the GSDMs require the specific and coordinated proteolytic processing by caspases for subsequent activation and pore-forming function (59, 60). In this regard, it is possible that such a mechanism could also exist to coordinate proper MPEG1 function.

## MPEG1 Structure and Mechanism

**Figure 3:**
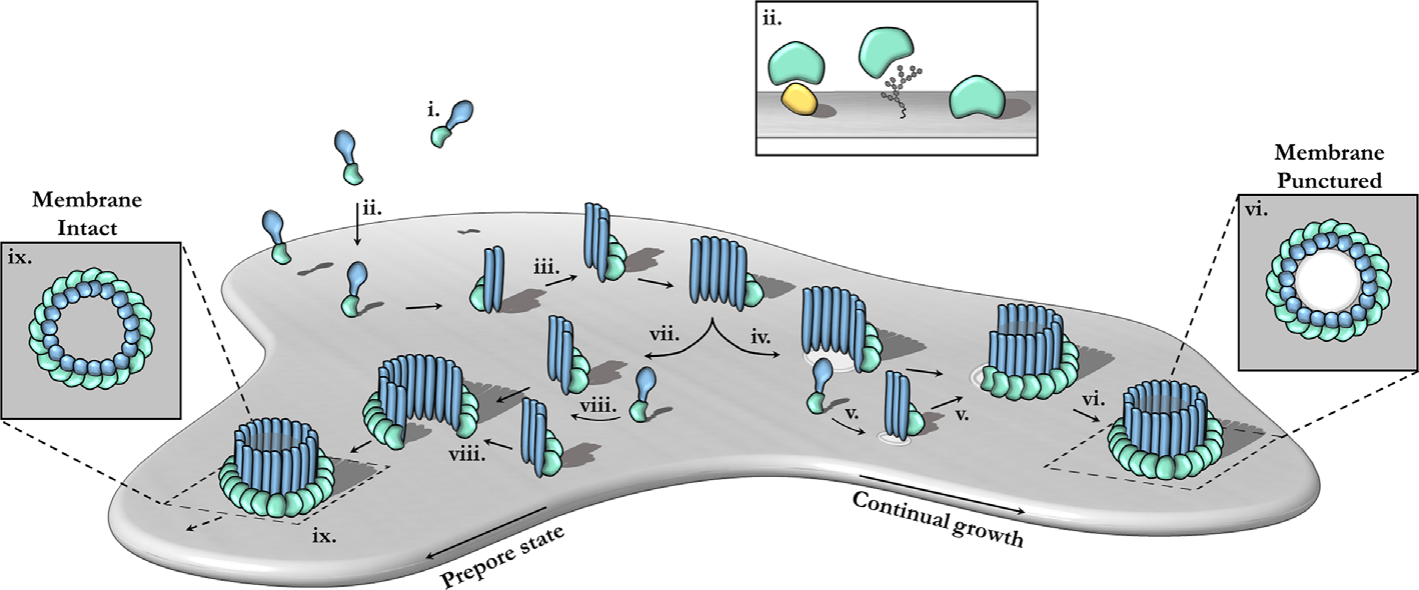
The canonical pathway of pore formation. A generic pore forming protein is shown, with a green ancillary (or receptor binding) domain and blue pore forming domain. Freely diffusing monomers **(i)** bind to the target bilayer (gray) **(ii)** *via* target recognition domains (green) that are ancillary to the pore forming machinery (blue). The target receptor can be proteins (yellow), glycans or lipids (**ii**; inset). Membrane-bound monomers undergo two-dimensional diffusion, colliding and eventually oligomerizing **(iii)**. Maturation *via* the prepore-to-pore conformational change may occur at different stages. For example, incomplete oligomers may transition into arc-pores **(iv)**. Other smaller arcs or monomers may also be recruited to a growing arc pore **(v)**. Ultimately complete pores are formed upon the closure of the oligomeric ring **(vi)**. In this context pore growth can occur in a continuous mechanism. Completed pores define large aqueous channels, capable of facilitating the passive diffusion of additional effector molecules (not shown) *via* the membrane channel (**vi**; inset). Alternatively, arc prepores may continue to grow without inserting into the membrane **(vii)** by recruiting additional monomers or other smaller arcs **(viii)**. These can ultimately form complete prepores that have yet to punch into the lipid bilayer **(ix)**. Fully formed prepores are most commonly observed for CDCs **(ix)**. The prepore-to-pore transition is triggered resulting in a conformational change of the MACPF core machinery that unfurls into a giant *β*-barrel (**ix** goes to **vi**). These inserted pores possess an amphipathic region that is fully inserted into the lipid membrane (not shown). Insets **(ix**, **vi)** show top-down views.

Pore-forming MACPF proteins generally adopt two different stable conformations—a soluble, metastable monomeric form and a membrane inserted hyper-stable oligomeric pore form (**Figure 3**). The pathways from the metastable monomer to the stable oligomer pore can vary. These topics have been extensively reviewed elsewhere in detail (15, 17, 18, 58, 98–102).

### Structural Biology of the MACPF Domain

**Figure 4:**
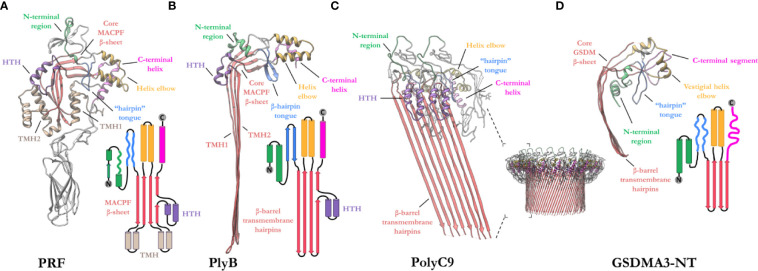
Exemplar structures of MACPF pore forming proteins in the monomeric and pore states. **(A)** Crystal structure of lymphocyte PRF in the monomeric state [PDB: 3NSJ] (43). The ancillary domain is colored gray and omitted from the topology diagram for clarity. **(B)** The cryoEM pore structure of the fungal MACPF protein, pleurotolysin (PlyB)[PDB: 4V2T] (13). Pleurotolysin is a homolog of PRF found in oyster mushroom. The *β*-trefoil domain of PlyB is not shown for clarity. **(C)** A dimer of the polyC9 cryoEM reconstruction (bottom right) is shown to illustrate the intra-subunit contacts at the MACPF interface [PDB: 6DLW] (22, 23). **(D)** The cryoEM pore structure of the intracellular GSDMA3-NT shows structural and topological similarity to the MACPF domain [PDB: 6CB8] (103). HTH, helix-turn-helix (purple); TMH, transmembrane *β*-hairpin (pale brown). Topology diagrams are colored consistently with the PDB coordinates.

The MACPF domain has two well documented functions; firstly, oligomerization into rings and, secondly, insertion into membranes. The domain is a well characterized fold that is centered around a contorted four stranded antiparallel *β*-sheet that features an L-shaped bend (**Figure 4**). The *β*-sheet is flanked at one end by two clusters of *α*-helices, termed transmembrane *β*-hairpin-1 (TMH-1) and TMH-2 (**Figure 4A**). The term, seemingly a misnomer, refers to the end-state conformation of these microdomains. Structural comparisons reveal that the MACPF domain undergoes a dramatic concerted conformational transition (104). These two bundles of *α*-helices (in the monomeric form) undergo structural rearrangement, unfurling to form a set of amphipathic *β*-hairpins (in the final pore form) (13, 21, 22, 83, 105) (**Figures 4A, B****)**. As they unwind the TMH regions are postulated to concurrently zipper up into *β*-hairpins, these protrude below the MACPF domain and thereby form a giant, amphipathic *β*-barrel that inserts into the membrane. Together these two *β*-hairpins each contribute four *β*-strands to the final oligomeric *β*-barrel pore (**Figure 4C**). From the perspective of the MACPF domain, the initial membrane associated oligomerization event includes the formation of *β*-sheet hydrogen bonds between the central *β*-sheets of adjacent subunits to form a nascent *β*-barrel. During the prepore-to-pore transition, the unfurling of the TMH regions is also accompanied by a straightening of the central MACPF *β*-sheet that permits more substantive *β*-sheet hydrogen bonding between adjacent subunits (**Figures 4A, B****)** (13). A short helix-turn-helix (HTH) region sits on top of TMH1 and forms additional inter-subunit interactions (**Figures 4A, C****)**. This region is observed to shift during the prepore-to-pore transition in some studies (13, 22). A second larger “helix elbow”, which contains the MACPF consensus motif (Y-X(6)-[FY]-G-T-H-[FY]), protrudes away from the core *β*-sheet and forms contacts around the periphery of the complex (**Figures 4A, C****)**. This helix elbow forms a complementary surface to accommodate TMH2 (**Figure 4A**). Outside the MACPF domain, studies on C9 and MPEG1 reveal that extensive inter-subunit interactions are also formed by the ancillary domains N- and C-terminal to the MACPF domain (23, 82). These interactions aid in the formation of oligomers (**Figure 4C**). The ancillary domain varies substantially between superfamily members, but its function is typically associated with membrane binding or targeting (15, 17, 100, 101).

A similar fold, from the GSDM family has strong topological parallels to the MACPF domain (**Figure 4D**). Indeed, DALI analysis revealed the pore-forming domain of GSDMs is a *bona fide* homolog of MAPCFs (62). Like MPEG1, GSDMs are regulated by proinflammatory signals and rely on proteolytic processing for activation (106). While the two families appear substantially different, GSDMs and MACPFs share mechanistic similarities—namely PRF, MAC, and GSDMs assemble and form transmembrane pores in a “growing-pore” like the model discussed below (20, 63, 94). Additionally, both families share topologically equivalent transmembrane *β*-hairpins which contribute to a giant *β*-barrel (86, 104, 107). The relation between the two families, however, still remains controversial.

### Overview of the MACPF Mechanism of Pore Formation

In the archetypal pathway of pore assembly, soluble monomers (**Figure 3i**) are generally recruited to the membrane surface *via* the function of domains that are ancillary to the MACPF domain and that function to directly bind to lipids or membrane associated protein receptors (**Figure 3ii**). In the monomeric form, the pore forming MACPF domain is folded into a compact structure that represents a state of high potential energy—it is primed and ready to punch into a lipid bilayer, akin to a compressed spring (**Figure 4A**). Membrane-bound monomers then undergo two-dimensional lateral diffusion and oligomerize to form prepore oligomers perched above the target membrane (**Figure 3iii**). These prepores are short-lived intermediate complexes comprised of rings or arcs that are yet to insert into the bilayer (83, 94, 104). These arciform or complete prepores can undergo the MACPF prepore-to-pore transition and insert into the lipid membrane (**Figures 3iv–vi**). Both membrane-inserted and uninserted arcs can continue to grow by recruiting subunits (**Figures 3iv–vi**). Both the mechanisms of prepore-to-pore transition and how prepores are triggered to form pores remain to be fully understood. In the final structure, each monomer contributes two amphipathic *β*-hairpins, and the final pore comprises a giant membrane spanning *β*-barrel (23, 43, 86, 104, 108). The oligomeric pore form represents the final, highly stable, membrane inserted state of the MACPF domain (**Figure 3vi**).

The archetypal MACPF mechanism described above was originally derived from extensive studies of the bacterial CDC branch of the superfamily (100, 104, 109, 110) (**Figures 3vii–ix**). However, in the context of MACPF proteins it is clear that numerous variations of this mechanism have been identified. Most importantly the concept of a prepore is less applicable to the MACPF branch of the superfamily. For example, the complement MAC forms a hetero-oligomer that assembles by recruitment of multiple different MACPF domain-containing subunits (C6, C7, C8, and C9) (21, 105). The MAC is also distinct in that membrane insertion into the target bilayer most likely takes place in a sequential, non-concerted manner with individual monomers progressively undergoing conformational change and membrane insertion one-by-one (20, 22). In addition, the MAC lacks membrane recognition domains, instead it is recruited to the target membrane *via* opsonization and the formation of complement component 5b on the surface of pathogenic microbes. Studies on PRF also reveal variations from the archetypal CDC-like mechanism—most notably the “prepore” form of PRF oligomers is highly mobile and flexible (94). PRF is thought to assemble and insert into the membrane in a growing pore model—where subunits or smaller arcs are recruited to a growing membrane inserted form (94, 111). PRF appears to only form ordered ring-like structures upon transition to the final pore form (94). Deviations of the common mechanism also are evident in pleurotolysin and perivitellin-2, which are two-component toxins (12, 13).

### Three-Dimensional Structure of MPEG1

Two studies have reported high resolution structures of truncated MPEG1 (82, 83). These studies employ MPEG1 constructs that lack the transmembrane and cytosolic regions, under the assumption that proteolysis of MPEG1 *in vivo* results in the same final primary sequence. These structures thereby represent presumed late matured MPEG1. These discoveries have propelled our understanding with respect to MPEG1 biology.

#### Membrane-Bound Prepore

**Figure 5:**
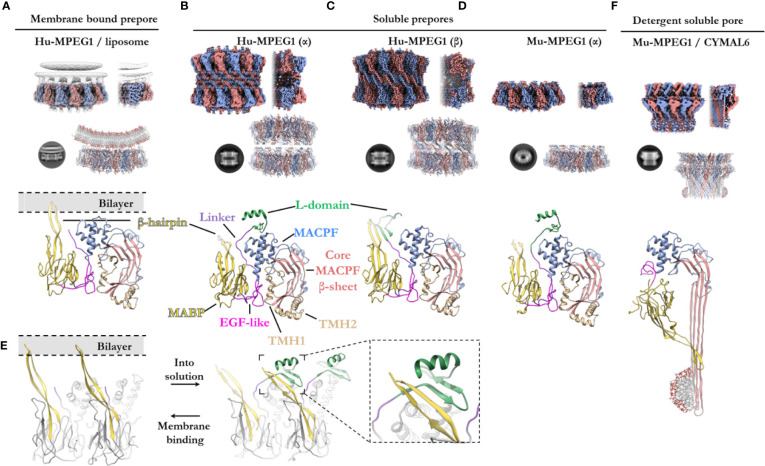
The various structural states of MPEG1. **(A)** When incubated with liposomes (gray), MPEG1 binds the lipid bilayer as a single prepore ring, *via* the MABP *β*-hairpin (yellow), orienting the MACPF domain away from the lipid bilayer [PDB: 6U2W] (82). Lipids are illustrated with a cartoon model. Both the unsharpened (gray) and sharpened (alternating color) maps are superimposed to illustrate the lipid density (gray). **(B)** In solution, recombinant MPEG1 (truncated between the L-domain [green] and TM region [not shown]) forms a loosely associated ring–ring dimer whereby the helix of the L-domain mediates interactions between rings (termed the *α*-conformation) [PDB: 6U2J, 6U2K] (82). **(C)** A second, tightly associated ring–ring dimer is also possible; this conformation is defined by inter-ring strand swapping (termed the *β*-conformation) [PDB: 6U2L] (82). This is achieved by the L-domain which adopts an extended *β*-sheet conformation. **(D)** Murine MPEG1 truncated at a similar position to **(A, B)**, forms single ring structures after prolonged incubation in acidic conditions [PDB: 6SB3] (83). These rings were observed in the *α*-conformation (with respect to the L-domain). **(E)** A view of an MPEG1 dimer is shown from the periphery of the complex in both the membrane-bound (left) and soluble prepore (right) states. Upon interchanging between these states, the L-domain and *β*-hairpin undergo conformational change. Inset shows a magnified view of the interaction. **(F)** Incubation of murine MPEG1 at low pH and in the presence of the detergent CYMAL6 results in MPEG1 pores [PDB: 6SB5] (83) where the MABP domain is flipped relative to **(A–D)**. This conformational change re-orients the MACPF and MABP domains into the same direction. The extremity of the *β*-barrel forms an amphipathic region (illustrated by a cartoon micelle). Top row: CryoEM reconstructions of MPEG1 (alternating colors show individual subunits) of the overall quaternary structure. Both the full reconstruction (left) and a partial cross section (right) are shown for each panel **(A–D, F)**. The cross section enables visualization of the inner structure of the complex. Second row: Exemplar 2D class averages are shown below each reconstruction [reproduced from (82, 83)]. The atomic coordinates for the full reconstruction are shown next to the corresponding 2D class average (alternating colours show individual subunits). Third row: A single magnified subunit from each complex is shown. MACPF *β*-sheet (red), TMH regions (tan), MACPF core (blue), MABP/*β*-hairpin (yellow), EGF-like (pink), linker region (purple), L-domain (green).

Overall, the ultrastructure of MPEG1 strongly reflects other pore forming proteins (**Figures 5A–E**). The membrane-bound structure of the MPEG1 ectodomain exists as a hexadecameric homo-oligomer, whose central core consists of the MACPF domain in a yet-to-unfurl, prepore state (**Figure 5A**). The MACPF core *β*-sheet is oriented such that release of the TMH helices would result in a *β*-barrel pore in an adjacent membrane.

The MABP domain of each subunit is positioned around the periphery of the central MACPF core. An extended, twisted *β*-hairpin protrudes from the MABP domain, in the opposite direction to the MACPF *β*-sheet and TMH regions, to make contact with the lipid membrane. All sixteen subunits coordinate the lipid bilayer in this manner and act to adhere the MPEG1 prepore to the membrane surface (**Figure 5A**). The hydrophobic tip of the MABP *β*-hairpin is surrounded by positively charged residues that together interact with the lipid bilayer and charged head groups of phospholipids (82, 83). This binding mode was observed for the canonical MABP domain and suggests the *β*-hairpin is an elongated variant of the similarly charged membrane-binding loop of the ESCRT MABP (85).

The L-domain (CTT domain) is located in close proximity to the lipid bilayer and, in the primary sequence, directly precedes the transmembrane helix and cytosolic tail (**Figure 1A**). However, in the membrane-bound prepore structure the L-domain is unresolved and, therefore, is presumed to be disordered (not shown).

#### Prepore (Soluble)—Role of the L-Domain

*In vitro* studies of the MPEG1 ectodomain have demonstrated that MPEG1 can adopt various conformational states (82, 83). Human MPEG1 was seen to form dimeric ring/ring complexes that are loosely associated (denoted the *α*-conformation) (**Figure 5B**). Some of these dimeric complexes were observed to undergo inter-ring, *β*-strand swapping to form tightly associated double-ring complexes (denoted the *β*-conformation) (**Figure 5C**). These different interactions are mediated by the L-domain and, hence, it was named due to its labile (L) conformational states. It is unclear whether the double-ring assemblies of human MPEG1 exist *in vivo* and it is suggested they likely represent *in vitro* artefacts (82). Unlike human MPEG1, murine MPEG1 was observed to form monomeric material that readily oligomerized to form single rings on membrane bilayers or in solution after prolonged incubation at pH 5.5 and 37°C (**Figure 5D**). With the exception of the *β*-conformation, both murine and human soluble MPEG1 complexes are very similar overall.

Comparison between the soluble and membrane-bound prepore conformations reveals relatively few differences, with one notable exception. In the soluble prepore (*α*-conformation; **Figures 5B, D****)**, the *β*-hairpin of the MABP domain interacts with the L-domain of the neighboring subunit (**Figure 5E** [right]). Due to this interaction, the *β*-hairpin is shifted relative to the membrane-bound prepore (**Figures 5D, E**[left]). These interactions stabilize the L-domain, which forms a small *β*-hairpin motif capped by an *α*-helix (**Figures 5B, D****)**. The *β*-hairpin and L-domain interaction creates a four strand *β*-sheet (**Figure 5E****; inset**), and hence these inter-subunit contacts anchor adjacent subunits in place. These interactions presumably provide stability to the complex and, therefore, were suggested to mediate oligomerization in solution. Truncation of the L-domain was reported not to affect membrane binding, however oligomerization in solution was not reported (83).

Therefore, in order for the soluble prepore to bind membranes it must undergo a conformational change. To accommodate the interactions with the membrane bilayer the MABP *β*-hairpin in the soluble prepore must bend upward ~25° and shift laterally away from the L-domain (**Figure 5E**). This movement breaks the intermolecular interactions with the L-domain of the adjacent subunit and positions the *β*-hairpin to interact with lipid head groups. In the absence of the interactions with the *β*-hairpin, the L-domain of the adjacent subunit becomes flexible or disordered. Furthermore, movement of the L-domain helix is necessary to accommodate the bending of the *β*-hairpin, which would otherwise produce steric clashes.

#### Detergent Solubilized Pore

Recently Ni and colleagues reported a structure of the murine MPEG1 pore (**Figure 5F**) (83). Incubation of murine MPEG1 in mildly acidified buffer led to oligomerization in solution, with further incubation in strongly acidified buffer (pH 4.0–3.6) promoting pore formation. Performing these reactions in the presence of the detergent, Cymal-6, stabilized the exposed hydrophobic regions of the *β*-barrel for structural studies. As expected, these experiments revealed that, like polyC9 and CDCs, the MPEG1 TMH regions unfurl to form giant *β*-barrels (**Figure 5F**).

Strikingly, the MPEG1 pore revealed significant structural rearrangement of the MABP domain relative to the MACPF domain (**Figures 5D, F****)**. The large rotation of the MABP domain results in the membrane-binding *β*-hairpin region oriented in the same direction with the *β*-barrel of the MACPF domain. Unlike most MACPF mechanisms studied to date, the peripheral ancillary domains typically do not undergo such drastic conformational changes, and hence these data suggest MPEG1 adopts an entirely unique mechanism. It remains unclear how and when this conformational change occurs, *i.e.* from a structural perspective and at which point of the MPEG1 assembly pathway. Higher resolution structures, as well as studies in the presence of lipid bilayers, will be required to fully understand the mechanism and structural motifs that mediate this unique transition.

### Control and Regulation of Oligomerization

**Figure 6:**
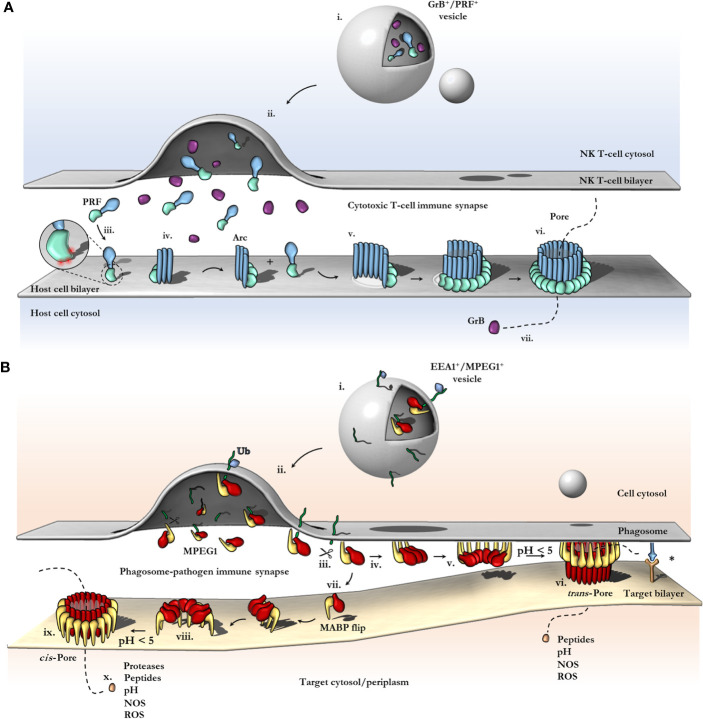
Schematic comparison between current models of PRF and MPEG1 assembly. **(A)** At the T-cell immune synapse, vesicles (i) containing PRF [only the MAPCF (blue) and C2 domains (green) are shown] and granzymes (purple) fuse with the plasma membrane releasing their contents onto the target cell (ii). Within these cytotoxic granules (i), PRF is kept in a Ca^2+^-deficient environment at low pH, therefore PRF is unable to bind membranes. Upon being released into the immune synapse (ii), PRF encounters Ca^2+^ (zoomed inset; red spheres) and therefore, binds the lipid bilayer *via* a target recognition C2 domain (iii). PRF begins to oligomerize into arcs (iv) and, later, fully formed pores (vi). In the PRF mechanism, early arc intermediates can puncture the lipid membrane (v); these can continue to grow in a continuous manner by recruiting monomers or other arcs. Functional arcs that have punctured into the lipid bilayer are depicted with a white membrane lesion (v). The final PRF pore enables granzyme B (GrB; purple) to diffuse into the target cell (vii). **(B)** EEA1^+^ vesicles containing MPEG1 (i) are triggered to traffic toward and fuse with the phagosomal membrane by monoubiquitination (blue diamond) (ii). Tethered MPEG1 [only the MACPF (red) and MABP domains (yellow) are shown] is proteolyzed from the lipid bilayer [transmembrane region and cytosolic tail are shown as a line (green)] (iii). Cleaved MPEG1 oligomerizes into a prepore (v). Upon strong acidification (pH < 5), MPEG1 is activated and transitions into a pore (vi or ix). MPEG1 may follow two proposed pathways (iv or vii). In the trans-pore model, oligomerization occurs on the host bilayer (iv to v) and *trans*-pores breach the bilayer of target membranes in close proximity (vi) (82). Other receptor complexes may be required to drive the formation of a close membrane–membrane junction (blue/orange receptor complex; asterisk). Alternatively, MPEG1 monomers diffuse within the synapse (vii) and oligomerize on microbial bilayers (vii to viii). The MACPF or MABP domains rotate, to re-orient the MACPF machinery toward the microbial bilayer [vii or viii; unclear (83)]. A *cis*-pore breaches the microbial bilayer (ix). The stage of MACPF or MABP domain rotation is unclear. After either a *trans*- or *cis*-pore has formed, effector molecules enter the target cell *via* the MPEG1 pore (x).

In most PFPs studied to date, oligomerization on the target bilayer results in a rapid trajectory into a final lytic pore. In this regard, the correct spatiotemporal control of oligomerization represents a key regulatory mechanism of PFP activity and safeguards the host cell from premature lytic activity. For example, in the PRF system, storage of PRF monomers within acidified vesicles prevents key aspartic acid residues in the C2 domain from chelating Ca^2+^, which prevents the C2 from adopting the membrane binding conformation (112). Therefore, PRF cannot bind membranes and does not oligomerize within granules. Subsequent release into the junction of the immune synapse causes a pH shift, and Ca^2+^ binding is restored, thus PRF binds the cell plasma membrane and rapidly oligomerizes to form pores (**Figure 6A**). During off-target assembly of the MAC on host cells, the inhibitor CD59 directly binds the nascent growing MAC and therefore blocks subsequent recruitment of C9 monomers, halting further oligomerization and safeguarding the cell (113). Notably, patients who lack CD59 develop PNH due to uncontrolled MAC activity (37). Given the intracellular context of the MPEG1 system, it is unclear what triggers oligomerization and when it occurs. Like the MAC and PRF systems, controlling MPEG1 oligomerization may act to prevent premature lytic activity that may have detrimental effects on the cell. In this regard, the coordinated cellular re-distribution of MPEG1 is critical in ensuring MPEG1 is appropriately located and poised to encounter microbes. However, cellular redistribution may additionally function to control the oligomerization event itself. As discussed earlier, proteolysis is suggested to regulate MPEG1 oligomerization (79). Moreover, sufficiently low pH was observed to be important for murine MPEG1 oligomerization (83). Thus, delivery of MPEG1 to acidified vesicles that possess the appropriate proteases may represent an additional level of functional regulation.

### Role of MABP *β*-Hairpin for Membrane Association

In agreement with structural data, independent studies found the MABP *β*-hairpin directly mediates lipid binding *in vitro*, preferentially recognizing negatively charged phospholipids (phosphatidylserine, cardiolipin, phosphatidylinositol(s), *E. coli* lipid extract, LPS) but not neutrally charged lipids (phosphatidylcholine, phosphatidylethanolamine, sphingomyelin) (82, 83). Therefore, consistent with other MACPF superfamily members (13, 43, 102, 104), the ancillary domain of MPEG1 appears to function for membrane targeting.

In all other MACPF systems studied to date, both assembly of the pore and perforation of the bilayer occurs on the same membrane, *i.e.* the assembly and target membrane are the same (13, 20, 21, 43, 94, 105, 114) (**Figure 6A**). In contrast, the MPEG1 assembly pathway may not follow this simple concept (**Figure 6B**). As discussed, the MABP and MACPF domains are functionally positioned in opposite directions. As such the MPEG1 system was observed to form soluble oligomers that were capable of diffusing and binding membranes (orienting the MACPF domain away from the bilayer). Therefore, the assembly membrane may not correspond to the target membrane.

Pang, Bayly-Jones and colleagues suggest this may function as a control mechanism to prevent MPEG1 oligomers from mediating autolysis (*i.e.* of the assembly membrane) (82). While the MABP *β*-hairpin is associated with the endosomal host bilayer (assembly membrane) (**Figures 6B iv–v**), the orientation of the MACPF domain would prevent membrane perforation of the host bilayer. Hence, these data suggest the MABP *β*-hairpin may act to sequester MPEG1 to the host bilayer and orient it in a protective capacity.

Conversely, *in vitro* high speed AFM imaging suggests these prepores could undergo a conformational change upon acidification that can be interpreted as pore formation (83). These experiments suggest that, rather than a protective role, the MABP *β*-hairpin may perform a targeting role as an ancillary domain in a more typical MACPF/CDC sense (**Figures 6Bvii–viii**). However, in this context the assembly of the prepore occurs independently from the target membrane (either in solution or on another bilayer)—this ability itself is unique when compared to other family members.

These features suggest that MPEG1, unlike other MACPF/CDC systems studied to date, may follow a distinct assembly pathway with independent assembly and target membranes (regardless of the role of the MABP *β*-hairpin). Both the ability to form soluble active prepore oligomers and the unique domain arrangement distinguish MPEG1 from other characterized MACPF/CDCs. Indeed, the canonical mechanism of pore formation and the notion of a single assembly/target membrane may not be consistent with the MPEG1 system.

### Acid Induced Pore Formation

Recent structure–functions studies reveal MPEG1 activity is strictly dependent on acidification, with recombinant MPEG1 becoming increasingly active at lower pH, with pH 5.5 representing an upper limit for detectable activity (82, 83) (**Figure 2ix**). How exactly pH triggers the prepore-to-pore transition of MPEG1 is not understood. The motifs and residues that mediate this acid trigger have not yet been established. Inspection of the core domain of MPEG1 does not reveal titratable residues that clearly govern key interactions for activation. As an alternative hypothesis, perhaps the low pH has an overall destabilizing effect and thus reduces the necessary activation energy. In any case, the acid trigger represents an important negative regulatory mechanism employed by the cell. This mechanism also explains why MPEG1 activity occurs within the matured endosomal vesicles and the phagolysosome. Furthermore, inhibition of endosomal acidification, for example by inhibitors of vacuolar-type H^+^-ATPases, would likely confer resistance to intracellular microbes by preventing the activation of MPEG1. This is a known mechanism of microbial evasion and is described for several pathogens, reviewed elsewhere (115). In contrast, earlier studies of MPEG1 function examined endosomal pH levels and found vesicles occupied by the intracellular pathogen *L. monocytogenes* rapidly acidify in MPEG1 deficient cell lines (116). Conversely, acidification was significantly delayed when MPEG1 expression was rescued. These data suggest that MPEG1 somehow functions to slow the rapid acidification of these phagosomal vesicles. These observations appear paradoxical. Specifically, it is unclear how exactly MPEG1 functions to reduce the acidification of vesicles *in situ*, while being strictly dependent on low pH for activity *in vitro*.

### MPEG1 Proposed Models and Mechanisms

The archetypal model of pore formation has largely arisen to describe PRF and CDCs (**Figure 3**); however, this canonical mechanism fails to explain how intracellular MACPFs operate within membranous compartments without detrimental effects to the host cell. Furthermore, membrane tethered MACPF systems, such as MPEG1 and ASTN1/2, challenge the notions of diffusion, membrane recognition, and oligomerization; in particular, the sequence of events may be different or additional steps may be required, such as proteolysis. There are currently two proposed models of pore formation, both of which may co-exist depending on context, together these represent an exciting frontier in understanding MPEG function (**Figure 6B**). In this regard, *in situ* data, such as cryo-electron tomography (cryoET) of MPEG1 in cells, will be important to determine the final pore state, mechanism of bactericidal activity and whether the prepore observed thus far represents a productive intermediate on the way to pore formation.

#### The Immunosynapse and *Trans*-Pore Formation

The advances in MPEG1 biology highlighted by recent studies address a critical question of how an intracellular MACPF can function within membrane compartments without killing the host cell. Specifically, the orientation of membrane-bound MPEG1 suggests pore formation can occur in an adjacent membrane (**Figure 6B vi**). As a result, MPEG1 may bridge two bilayers; one membrane is coordinated by the MABP *β*-hairpin, while the other becomes the target of the MACPF *β*-barrel of the MPEG1 pore (**Figure 6B vi**). Such a mechanism is akin to a primordial (intracellular) immune synapse, where the phagosomal membrane and target membrane are brought into close proximity by conjugate cellular mechanisms *e.g.* immune receptor:antigen interactions (**Figure 6B**; right). Such proximity would then allow for MPEG1 pores to preferentially damage pathogen bilayers, while providing a method of protecting the host bilayer. Of course, to ensure that pore formation results in damage to a target membrane, it is important that acidification occur after a synapse has been established such that membranes are in sufficient proximity. Importantly, these *trans*-pores were observed in cryo-EM data sets of MPEG1 proteoliposomes (82); however, these were a rare population, and 3D reconstructions were not possible. Further structural and biochemical studies will be required to confirm or exclude their existence.

A key implication of the *trans*-pore model is that MPEG1 has little target specificity, rather MPEG1 would damage any membrane that is sufficiently close upon activation. Similarly, broad anti-membrane activity is thought to occur for the MAC, which employs an atypical target-recognition mechanism (20–22, 105). Likewise, upon its delivery, PRF must recognize any host cell membrane in the immune synapse. In this regard, PRF has been shown to form pores on several lipid compositions, relying more specifically on the physical properties of lipid fluidity to distinguish its target (117) (**Figure 6A**). An evolutionary advantage would be conferred by immune effectors that can broadly target and damage a variety of membrane compositions and thus a range of pathogens. Therefore, metazoan immune complexes such as MPEG1, MAC, and PRF may have evolved greater flexibility in their target recognition, while relying more heavily on conjugate cellular mechanisms to govern their regulation *e.g.* PRF granules, or the MAC-inhibitor CD59 (118) or the domain arrangement in MPEG1 (82, 83). Broad anti-membrane activity would maximize the spectrum of potential vulnerable targets; however, it would also simultaneously make the organism more susceptible to collateral damage. Therefore, conjugate cellular mechanisms might be more important in governing the regulation, delivery, and activation of the mammalian perforin-like immune effectors (**Figure 6A**). The promiscuity of these immune effectors is highlighted when juxtaposed against other PFPs that have highly specific target recognition requirements (*e.g.* pleurotolysin, intermedilysin) (13, 119).

#### MABP Rotation and *Cis*-Pore Formation

In contrast, the recent structure of a murine MPEG1 pore at 5 Å illustrates an unexpected conformational rearrangement of the MABP domain (83) (**Figure 6B viii**). Comparison of MPEG1 structures as a soluble prepore and a detergent solubilized pore suggests a different model of pore formation occurs, whereby rotation of the MABP domain and *β*-hairpin motif is responsible for recognition of the target membrane (**Figure 6B vi**). The drastic conformational rearrangement corresponds to a lateral 180° rotation of the MABP domain (such that the peripheral region remains on the periphery), resulting in the MABP *β*-hairpin and MACPF domains being re-oriented in the same direction (**Figure 5E**, **Figure 6B viii**). It is not clear whether this rearrangement would occur prior to acid-induced pore formation or accompany pore formation. One possibility is that the MABP *β*-hairpin swings down to recognize the target membrane, engaging the bilayer *via* the lipid binding motif (**Figure 6B vi**). Subsequently, upon acidification, the MPEG1 prepore (here in a late stage) unfurls to form a giant *β*-barrel in the target. In support of this model is a disulfide trapped mutant, which locks the MABP domain to the linker region, preventing domain movement. This mutant loses lytic activity until a reducing agent is added, restoring activity to that of wild type. These data suggest that MPEG1 activity is dependent on the movement of the MABP domain.

While there are some parallels between this model and the archetypal mechanism, the model is nevertheless unique. Specifically, MPEG1 may adopt two prepore states, both an early stage (either soluble or membrane-bound; **Figure 6B iv**) and a late stage (MABP and MACPF in the same orientation; **Figure 6B vii**) [also depicted in Ni et al. (83)]. Furthermore, this model provides a simpler explanation of target recognition, without postulating coupled processes or receptors, as the MABP *β*-hairpin alone would be sufficient. Problematically, the *cis*-pore model fails to explain how freely diffusing MPEG1 could function within a membrane compartment without damaging the cell’s own membranes. A similar conundrum exists for PRF, which functions at the immunosynapse between a host and target cell (**Figure 6A**). One recent study suggests that differences in the fluidity of the lipid bilayer of cytotoxic T cells, compared to that in target cells, can protect T-cell membranes from PRF pore formation (117). Therefore, potential differences in membrane properties of the host and pathogen may likewise provide selectivity in the MPEG1 system.

## Discussion

*In vitro* studies of MPEG1 have provided key biophysical and structural insights into the MPEG1 assembly pathway. Two models have emerged that attempt to explain the MPEG1 mechanism, namely the *cis*- and *trans-*pore models; however, there remain several questions that neither assembly model fully addresses (**Figure 6**). At this point in time the field cannot resolve both models. In this regard it is obvious that the MPEG1 system deviates from other MACPFs. Indeed, the MPEG1 system appears to adopt a unique mechanism of pore formation altogether. Further work is required such as characterization of other potential structural states, as well as higher resolution data of current models. Particularly, *in situ* studies would be beneficial to ascertain the stepwise events in MPEG1 function, *e.g.* data acquired by imaging pores within the cellular environment by cryoET.

One issue with the *trans*-pore model is the apparent redundancy of the MABP domain and the transmembrane helix. It seems mysterious that MPEG1 would evolve two independent mechanisms for anchoring to the host membrane (**Figures 6B iii, iv**). In contrast, the *cis*-pore model is consistent with a need for both a transmembrane helix and a secondary membrane recognition domain for targeting, namely the MABP domain. Matters are complicated by the role of proteolysis where it is hypothesized that cleavage of the transmembrane helix is necessary for oligomerization (**Figure 6B iii**). The *trans-*pore model encompasses the possibility of proteolysis, whereby the MABP *β*-hairpin functions in sequestering MPEG1. In contrast, in the *cis-*pore model, proteolysis raises the issue of host cell protection where membrane compartments become susceptible to collateral damage by freely diffusing MPEG1.

Nevertheless, there is structural and biochemical evidence to support a *cis*-pore model, where it is proposed the MABP domain rotates to contact the target bilayer (**Figures 6 vii–xi**) (83). However, it is unclear how such a conformational shift would be physically possible in an oligomeric form, since significant clashes with neighbouring subunits would prohibit the MABP rotation. Secondly, substantial energy would be required to dissociate all sixteen MABP *β*-hairpins from the bilayer to allow for MABP rotation. Assuming the MPEG1 complex does not dissociate from the bilayer (**Figures 6 iv–vi**), structural comparison of the membrane-bound prepore (**Figure 5D**) and the inserted pore state (**Figures 5E**) suggests that the point of rotation is about the MACPF domain rather than the MABP domain. However, rotation of the MACPF domain seems highly unlikely. This would require extensive interactions formed at the MACPF interface to break or require subunits to dissociate entirely to accommodate the large motion. Taken together, within an oligomeric state, rotation of either the MACPF or MABP domains is difficult to reconcile. One possibility is that structural rearrangement occurs prior to oligomerization, *i.e.* as monomers (**Figures 6B iv, vi**); however this is inconsistent with the observed lytic activity of pre-assembled oligomers (82, 83). Alternatively, it is possible that the observed *cis*-pore may be artefactual, resulting from detergent solubilization or partial denaturation at pH 3.6**–**4.0. Further studies will be required in order to address these issues and confirm if, and how, the MABP domain rotates in the presence of lipid bilayers and under more physiological conditions.

It is worth considering similarities between MPEG1—the most ancient pore forming MACPF protein described to date—and the GSDMs—a somewhat recently described family of pore forming proteins that drive programmed cell death *via* pyroptosis. Both are intracellular pore forming proteins that share a similar function that is pore formation in immunity. Our analysis further reveals these molecules clearly share the same overall core topological fold (**Figures 4A–C**) in agreement with others (62), and both MPEG1 and GSDMs require activation *via* proteolysis. These comparisons reveal that both MACPF proteins and GSDMs utilize common mechanistic features to form large *β*-barrel lined pores—*i.e.* oligomerization of a substantial number of monomers and the unwinding of two topologically equivalent regions within each monomer to form membrane spanning amphipathic *β*-hairpins (**Figures 4A–C**).

Despite these observations, sequence comparisons reveal no obvious sequence conservation between the two families. Furthermore, certain structural features of MACPF proteins (*e.g.* the HTH motif) are absent in GSDMs. Thus, some controversy exists with respect to whether the similarities between GSDMs and MACPF proteins have arisen through convergent or divergent evolution. Based on bioinformatic studies of other protein families (120), we, and others (62, 63), suggest that the structural, functional, and mechanistic data collectively present a reasonably compelling argument for a common ancestry between MACPF proteins and GSDMs. Such a relationship, if further supported (for example through identification and structural characterization of proteins that link the two families), may reveal intriguing new aspects of how members of the MACPF superfamily have been deployed and re-deployed throughout the breadth of the immune response.

Herein, we have discussed the role MPEG1 plays within the cellular environment and its involvement as an immune effector. While several features of MPEG1 have been elucidated, many new questions have correspondingly arisen. For example, few studies have been performed that investigate the short MPEG1 isoform (MPEG1b), and hence, its role is poorly understood. Overall, the structural advances to date will provide a strong foundation for future work by guiding experiments and *in situ* studies. Of particular interest will be the impact of more directed mutagenesis and targeted changes to MPEG1 domains and key structural elements, to ascertain what effects these have on phenotypes in cellular and animal models. In this regard, further studies will be highly informative in delineating the physiological roles of MPEG1 and its assembly.

## Author Contributions

Drafting of manuscript by CB-J, JW and MD. Figures made by CB-J. Critical review and editing of manuscript by BS and SP. All authors contributed to the article and approved the submitted version.

## Funding

CB-J acknowledges the support of the Australian Government RTP scholarship. JW is an Australian Research Council (ARC) Laureate Fellow (FL180100019) and an Honorary National Health and Medical Research Council (NHMRC) Senior Principal Research Fellow (APP1127593). He acknowledges the previous support of an Australian Research Council (ARC) Federation Fellowship. MD acknowledges the support of an ARC Future Fellowship (FT150100049) and ARC Discovery Project (DP180100040). We thank the staff of the Monash Ramaciotti Centre for Electron Microscopy, the Monash protein production and proteomics platforms, and the support of the MASSIVE supercomputer team.

## Conflict of Interest

The authors declare that the research was conducted in the absence of any commercial or financial relationships that could be construed as a potential conflict of interest.

## Abbreviations

MACPF, Membrane Attack Complex/Perforin; CDC, Cholesterol Dependent Cytolysin; MPEG1, Macrophage-expressed gene 1; MAC, Membrane Attack Complex; PRF, Perforin; ASTN, Astrotactin; BRINP, bone morphogenetic protein/retinoic acid inducible neural-specific proteins; MVB12, multi-vesicular body-12; MABP, MVB12-associated *β*-prism; PNH, paroxysmal nocturnal hemoglobinuria; SPECT, sporozoite microneme proteins essential for cell traversal; LPS, lipopolysaccharide; IFN, interferon; TNF, tumor necrosis factor; MRSA, Methicillin-resistant *Staphylococcus aureus;* LAMP, lysosomal-associated membrane protein 1; EEA1, early endosome antigen 1; ESCRT, endosomal sorting complexes required for transport machinery; MyD88, Myeloid differentiation primary response 88; NF*κ*B, nuclear factor kappa-light-chain-enhancer of activated B cells; EGF, epidermal growth factor; (Cryo-)EM, (cryogenic) electron microscopy; (Cryo-)ET, (cryogenic) electron tomography; BMDM, bone marrow derived macrophages; MEF, Mouse embryonic fibroblast.
